# Microbiological, lipid and immunological profiles in children with gingivitis and type 1 diabetes mellitus

**DOI:** 10.1590/1678-77572016-0196

**Published:** 2017

**Authors:** Cristiane DUQUE, Mariana Ferreira Dib JOÃO, Gabriela Alessandra da Cruz Galhardo CAMARGO, Gláucia Schuindt TEIXEIRA, Thamiris Santana MACHADO, Rebeca de Souza AZEVEDO, Flávia Sammartino MARIANO, Natália Helena COLOMBO, Natália Leal VIZOTO, Renata de Oliveira MATTOS-GRANER

**Affiliations:** 1 Universidade Estadual Paulista, Faculdade de Odontologia de Araçatuba, Departamento de Odontologia Infantil e Social, Araçatuba, São Paulo, Brasil; 2 Universidade de Campinas, Faculdade de odontologia de Piracicaba, Departamento de Diagnóstico Oral, Piracicaba, São Paulo, Brasil.; 3 Universidade Federal Fluminense, Faculdade de Odontologia de Nova Friburgo, Nova Friburgo, Rio de Janeiro, Brasil.

**Keywords:** Gingivitis, Children, Diabetes mellitus, Polymerase chain reaction, Cytokines

## Abstract

**Objective:**

The aim of this study was to compare the prevalence of periodontal pathogens, systemic inflammatory mediators and lipid profiles in type 1 diabetes children (DM) with those observed in children without diabetes (NDM), both with gingivitis.

**Material and methods:**

Twenty-four DM children and twenty-seven NDM controls were evaluated. The periodontal status, glycemic and lipid profiles were determined for both groups. Subgingival samples of periodontal sites were collected to determine the prevalence of periodontal microorganisms by PCR. Blood samples were collected for IL-1-β, TNF-α and IL-6 analysis using ELISA kits.

**Results:**

Periodontal conditions of DM and NDM patients were similar, without statistical differences in periodontal indices. When considering patients with gingivitis, all lipid parameters evaluated were highest in the DM group; *Capnocytophaga sputigena* and *Capnocytophaga ochracea* were more prevalent in the periodontal sites of DM children. “Red complex” bacteria were detected in few sites of DM and NDM groups. *Fusobacterium nucleatum* and *Campylobacter rectus* were frequently found in both groups. Similar levels of IL-1-β, TNF-α and IL-6 were detected in DM and NDM children.

**Conclusion:**

Clinical and immunological profiles are similar between DM and NDM children. The presence of *Capnocytophaga sputigena* and *Capnocytophaga ochracea* were associated with gingivitis in DM children.

## Introduction

Periodontal disease comprises a group of conditions that affects the gingiva, periodontal ligament, cementum, alveolar bone, and tissue structures that support the teeth. The predominant form of periodontal disease in children and adolescents is gingivitis^13^. There is no clear-cut age at which the gingival reaction to bacterial insult in children converts to that found in adults. However, there is a gradual increase in gingival activity from early childhood to adult age^11^.

The etiology of periodontal disease is complex. Some bacterial species are recognized as putative periodontal pathogens^26^. In particular, *Tannerella forsythia* (*Tannerella forsythensis*), *Porphyromonas gingivalis* and *Treponema denticola,* known as “red complex” pathogens*,* have been indicated for playing important roles in various forms of periodontal diseases^7,26^. *Campylobacter sp.*, *Prevotella intermedia/Prevotella nigrescens*, *Fusobacterium sp.,* members of the “orange complex”, are also related to periodontal breakdown as the secondary group of periodontal pathogens and the “green complex,” represented by the combination of *Eikenella corrodens*, *Capnocytophaga sputigena, C. ochracea, C. gingivalis, C. concisus* was considered primary colonizers and compatible with periodontal health^26^ *.* Cortelli, et al.^6^ (2009) detected high levels of *Campylobacter rectus* associated with periodontal health and *Prevotella intermedia* with the presence of inflammation. Rotimi, et al.^18^ (2010) showed that, except for *P. gingivalis*, periodontopathogens such as *A. actinomycetemcomitans*, *T. forsythia*, *P. intermedia* and *P. nigrescens* are relatively common findings in the oral cavity of children. Thus, the relationship between clinical parameters and the prevalence of several periodontal pathogens in children need to be studied in greater detail.

The pathogenesis of periodontal disease has been widely revised^16,17^ and there is a consensus that, although bacteria are essential, they are insufficient for the disease to occur^16^. Microbial challenge in the subgingival plaque modulates the host immune-inflammatory response in the periodontal tissues^17^. Macrophages and polymorphonuclear leukocytes, in response to the chemo-attractant effect of bacterial toxins, such as lipopolysaccharide (LPS), are activated to produce important inflammatory mediators, TNF-α, IL-1-β, IL-6, and other cytokines^29^. These mediators are responsible for periodontal breakdown, leading to the clinical signs and symptoms of disease^17^. A few studies evaluated the production of cytokines in children with gingivitis. Ulker, et al.^29^ (2008) found a correlation between high levels of IL-1β and TNF-α in gingival crevicular fluid of children and clinical signs of gingivitis.

Systemic inflammatory diseases, such as diabetes, alter the host environment, and are predicted to increase the patient’s vulnerability to gingivitis due to changes in the inflammatory response to microbial challenges^12^. Clinical studies have demonstrated that the presence of diabetes can be considered a risk factor for periodontal disease in childhood^19,31^. Individuals with diabetes mellitus have impaired neutrophil and macrophage functioning, altered collagen production, and exaggerated collagenase activity^12^, perhaps leading to the patient’s heightened inflammatory state, as interactions with advanced glycation endproducts (AGEs) have been shown to increase macrophage secretion of proinflammatory mediators^12^. Salvi, et al.^19^ (2010) found a high concentration of IL-1β in patients with type 1 diabetes when compared to healthy individuals. Snell-Bergeon, et al.^24^ (2010) evaluated 553 patients with type 1 diabetes mellitus and 215 healthy patients aged between 10 and 22 years and observed that high levels of IL-6 and other biomarkers were associated with the lipid profile and may collaborate with systemic complications in individuals with diabetes. These complications could increase the patient’s risk to develop severe periodontal disease^31^. There are few studies that have examined microbial colonization, immunological factors and gingival health during childhood. The aim of this study was to compare the prevalence of periodontal pathogens, systemic inflammatory mediators and lipid profiles in type 1 diabetes children (DM) with those observed in children without diabetes (NDM), both with gingivitis.

## Material and methods

### Study population

The study protocol was approved by the Ethics Committee of Antonio Pedro University Hospital (protocol 057/2010). Children with type 1 diabetes mellitus (DM) and children without diabetes (NDM), aged between 7 and 13 years, with mixed dentition, of both genders, and without distinction of race were selected for this study. Individuals without diabetes were recruited from the Pediatric Dentistry Clinic and children with diabetes from the database kindly provided by a local Diabetes Association. The diagnosis of diabetes was given by an endocrinologist. The exclusion criteria used for subject recruitment^2^ were: antibiotic prophylaxis for dental treatment, uncontrolled systemic diseases, immunological compromise, subjects who were wearing orthodontic devices, subjects who had been undergoing periodontal treatment 12 months before the beginning of the study, those who had been taking antibiotics within 6 months prior to the clinical examination, those with extensive caries lesions, individuals who were using an antiseptic solution during 3 months period and smokers. Parents or legal guardians were informed of the study and signed an informed consent form and completed an interview regarding the medical and dental histories of the children.

### Clinical measurements

The following clinical parameters were measured: caries index [(dmf/DMF – decay (d), missing (m) and filling (f) deciduous (dmf) and permanent (DMF) teeth)^6^, probing depth (PD), plaque index (PI)^23^ and gingival index (GI)]^10^, by two previously calibrated examiners (CD and GACGC), using a periodontal probe (PCPUNC 15) (Hu-Friedy, Chicago, IL, USA) at four sites (mesiobuccal, mid-buccal, disto-lingual, mid-lingual) per tooth. The following teeth were examined: all first permanent molars, all second deciduous molars, two upper permanent incisors and lower permanent incisors. Permanent teeth were fully erupted. The intra-examiner and inter-examiner agreement of the categorical variables (PI, GI) using the Kappa calculation, at tooth level, was 0.72 and 0.68, respectively. Reproducibility of continuous variables (PD) was 0.71 and 0.69, respectively, as examined by the intraclass correlation coefficient (ICC).

### Intraoral samples collection

Before the intraoral collection procedures, cotton rolls were applied to prevent contamination of the sampling area with other oral fluids. The supragingival biofilm was gently removed using sterile cotton pellets and subgingival biofilm samples were collected using sterile paper points (Tanari #30, Tanariman Industrial Ltda., Manacapuru, AM, Brazil), which were inserted to the depth of the gingival sulci for 60 seconds. This procedure was performed for each of the four sites previously selected (mesiobuccal sulci of three permanent molars and one permanent incisor, selected randomly or mesiobuccal sulci of four deciduous molars) and the paper points of each subject were inserted in a microtube containing 1 mL of Tris-EDTA solution (10 mM Tris–HCl, 0.1 mM EDTA, pH 8.0) on ice. Pooled biofilms were separated according to dentition (permanent or deciduous) for each patient. The samples were stored at −80°C until the analyses.

### Blood samples collection

Patients were asked to reduce the intake of fatty foods the night before collecting the blood samples. Blood samples were collected by a specialized professional from the peripherical vein (cubital fossa) of individuals who had an overnight fast. Samples were collected in vacuum collection tubes and sent to Raul Sertã Hospital Laboratory at Nova Friburgo/RJ for clinical analysis [fasting glucose levels (GL), glycosylated hemoglobin – (HbA1c), triglycerides (TRG), total cholesterol (TC), high–density lipoprotein (HDL), low-density lipoprotein (LDL), very low-density lipoprotein (VLDL) and total lipids (TL)] using specific kits (Gold Analisa, Belo Horizonte/MG). One tube was centrifuged (3000 rpm/10 min) and the blood serum was carefully collected, aliquoted and frozen at −80°C for immunological analysis.

### Bacterium-specific PCR

The subgingival samples were thawed, vortexed and centrifuged (10,000 rpm/10 min). After removal of the paper points and supernatant, samples were submitted to DNA extraction using a protocol described by Sardi, et al.^21^ (2011); bacterial molecular identification was carried out by Polymerase Chain Reaction method using a thermal cycler (TPersonal, Biometra, Germany). The bacterium-specific primer sequences used are listed in the correspondent references: *Aggregatibacter actinomycetemcomitans* (Aa)^5^; *Campylobacter rectus* (Cr)^2^; *Capnocytophaga ochracea* (Co)^5^; *Capnocytophaga sputigena* (Cs)^5^; *Eikenella corrodens* (Ec)^2^; *Fusobacterium nucleatum* (Fn)^28^; *Tannerella forsythia* (Tf)^2^; *Treponema denticola* (Td)^30^; *Porphyromonas gingivalis* (Pg)^18^; *Prevotella intermedia* (Pi)^2^; *Prevotella nigrescens* (Pn)^2^. PCR reactions were standardized for each primer using genomic DNA from strains of culture collections as a positive control and distilled water as a negative control. PCR amplifications were performed using 200 µM of dNTPs, 2.5 mM of MgCl_2_, 0.3 µM of each primer, 1.25 U of Taq DNA polymerase (Invitrogen, Brazil) and approximately 10 ng of genomic DNA, to obtain a volume of 25 µl. Thermal conditions of each primer was tested, following the initial pattern: DNA denaturation at 95^o^C for 5 minutes, 35 cycles at 95^o^C for 30 seconds, primer hybridization at 55^o^C-62^o^C (depending on the primer) for 30 seconds, extension at 72^o^C for 1 minute and finalizing the reaction at 72^o^C for 7 minutes^2^. The PCR products were separated by electrophoresis in 2% agarose gels and Tris-borate-EDTA running buffer. The DNA was stained with 0.5 ug/mL ethidium bromide and visualized under UV illumination (Pharmacia LKB-MacroVue, San Gabriel, CA, USA). Each gel received a 100 pb or 1 Kb DNA Ladder (Invitrogen, Brazil).

### ELISA assays

IL-1-β, TNF-α and IL-6 serum levels were determined by ELISA kits (R&D Systems, Minneapolis, USA), according to the manufacturer’s instructions. Serum samples were assayed at 1:10 dilutions and evaluated in duplicate. Biomarker quantification was performed using a microplate reader (Molecular Devices, Programa Versa Max). Results were reported as pg/mL.

### Statistical analysis

The statistical analysis was performed using SPSS Statistics 17.0 (IBM Inc., Chicago, IL, USA). The subject characteristics (gender, age, fasting glucose level, HBA1c level and dmf/DMF) were compared between DM and NDM using the Student’s t-test for quantitative variables and Mann-Whitney U test for qualitative variables. Clinical parameters were compared between DM and NDM using Mann-Whitney test, except for PD, which was submitted to Student’s t-test. The percentage of sites with the tested bacteria and data from the questionnaire were compared between DM and NDM applying the Chi-square test. Immunological and lipid profiles were assessed using Student’s t-test. The Pearson correlation test was applied to find positive associations between periodontal status and other clinical parameters (lipid, microbiological and immunological profiles). The differences were considered significant when p≤0.05.

## Results

### Clinical characteristics of the study population

Twenty-four children with type 1 diabetes mellitus and 27 children without diabetes participated in this study. No significant difference was observed between DM and NDM groups, considering gender, age and caries level evaluation (dmft/DMFT). Table 1 describes the characteristics of the study population (DM and NDM groups). Fasting glucose and HBA1c levels were statistically different between the groups, always with the highest values for the DM patients. There were no significant differences for PI and GI scores and all PD measurements when comparing DM and NDM for both dentitions, indicating that both groups had similar periodontal conditions. Questionnaires given to children and their parents provided information about the gingivitis history and dental care of patients, such as presence of gingival bleeding, halitosis, mouth breathing and tooth brushing habits. There were no differences between DM and NDM for these parameters evaluated. Table 1 also presents lipid and immunologic profiles of DM and NDM groups, considering children with gingivitis (p≥2). All lipid parameters evaluated were the highest in the DM group; however, statistical difference was observed only for HDL, TRG and TL. There were no statistical differences between DM and NDM for IL-1-β, TNF-α and IL-6 detected in the serum of children.

**Table 1:** Characteristics of the study population

		NDM	DM
General Characteristics			
Gender n (%)	Male Female	13 (48.1) 14 (1.9)	12 (50) 12 (50)
Age (in years) Mean±SD		9.62 ±1.86	9.45 ±1.69
Fasting glucose level (mg/dL) Mean±SD		78.7± 8.10*	101.74 ±40.64
HbA1c % (mmol/mol) Mean [SD]		4.42 ±0.61*	6.94±1.58
Children using insulin for more than 1 year n (%) dmft/DMFT		- 0.93/0.11	13 (54.16) 0.94/0.13
Periodontal status			
Deciduous teeth			
PI**		24.2 (63) 6.2	12.6 (4.1) 3.5
GI**		15.3 (0) 5.04	16.3 (6.25) 6.0
PS		1.41±0.49	1.34±0.29
Permanent teeth			
PI**		32 (19.4) 6.0	24.7 (25) 4.62
GI**		19.1 (12.5) 3.9	17.6 (8.33) 4.5
PS		1.41±0.50	1.48±0.48
Lipid Profile			
HDL		45.9 (8.4)*	54.4(15.5)
LDL		82.7(21.5)	106.4 (37.5)
TRG		66.9(27.0)*	78.9(53.3)
TC		142.5(27.7)	167.5(46.5)
VLDL		13.5 (5.6)	16.12 (10.4)
TL		389.0(78.1)*	490(143.5)
Immunological profile			
IL-1β		1.98 (0.29)	1.57 (0.31)
TNF-α		15.01 (2.91)	11.48 (5.73)
IL-6		2.18 (0.29)	1.26 (0.30)

NDM – children without diabetes / DM – children with type 1 diabetes mellitus

PI = Plaque index, GI = Gingival Index, PD = depth probing, HDL = high density lipoprotein, LDL = low density lipoprotein, TRG = triglycerides, TC = total cholesterol, VLDL = very low density lipoprotein, TL = total lipids (values in mg/dL).

* Statistical difference when compared NDM vs. DM, according to the Student’s t-test for quantitative data and Mann-Whitney test for qualitative data (p≤0.05).

** Mean (Median) standard error of percentage of scores ≥2

### Association between periodontal status and lipid and immunological profiles

Tables 2 and 3 show the associations between periodontal status and lipid and immunological profiles. When comparing the periodontal status and cytokine levels, TNF-α was correlated with PI for deciduous teeth (Pearson correlation, r=0.935, p=0.002) and with GI for deciduous teeth (Pearson correlation, r=0.772, p=0.042) in patients with diabetes. IL-6 was correlated with PI for deciduous teeth (Pearson correlation, r=0.922, p=0.003) in patients with diabetes. When considering the immunological and lipid profiles, IL-1β was positively correlated with LDL (Pearson correlation, r=0.696, p=0.037) TC (Pearson correlation, r=0.671, p=0.048) and TL (Pearson correlation, r=0.7181, p=0.029) for DM children and with TRG (r=0.444, p=0.044) for NDM children. IL-6 was positively correlated with HDL (Pearson correlation, r=0.791, p=0.011) for DM and LDL (Pearson correlation, r=0.501 p=0.021) for NDM.

**Table 2:** Associations between lipid and immunological results for children with gingival bleeding, considering NDM and DM groups

	Condition	glucose	HBA1c	HDL	LDL	TRG	TC	VLDL	TL
IL-1β	NDM	r=0.2701	r=0.1919	r=0.0079	r=-0.2876	**r=0.4436**	r=-0.1416	r=0.4119	r=0.0333
		p=0.236	p=0.405	p=0.973	p=0.206	p=0.044*	p=0.540	p=0.064	p=0.886
	DM	r=00.5333	r=0.2961	0.2561	**r=0.6958**	r=0.1580	**r=0.6710**	r=0.1815	**r=0.7181**
		p=0.139	p=0.439	p=0.506	p=0.037*	p=0.685	p=0.048*	p=0.640	p=0.029*
TNF-α	NDM	r=-0.0856	r=-0.2661	r=0.0426	r=0.2157	r=-0.0142	r=0.1473	r=-0.1148	r=0.0908
		p=0.712	p=0.244	p=0.855	p=0.348	p=0.951	p=0.524	p=0.620	p=0.695
	DM	0.3050	0.1283	0.5249	-0.0419	r=-0.3790	r=0.0818	r=-0.3780	r=-0.1065
		p=0.425	p=0.742	p=0.147	p=0.915	p=0.314	p=0.834	p=0.316	p=0.785
IL-6	NDM	r=-0.1447	r=-0.1661	r=0.0833	r=0.5008	r=0.1670	r=0.4177	r=0.0661	r=0.3702
		p=0.532	p=0.472	p=0.720	p=0.021*	p=0.469	p=0.060	p=0.776	p=0.099
	DM	r=0.1604	r=0.0771	**r=0.7914**	0.2441	r=-0.3838	r=0.3987	r=-0.3738	r=0.1642
		p=0.680	p=0.844	p=0.011*	p=0.527	p=0.308	p=0.288	p=0.322	p=0.673

* Positive correlations obtained by Pearson correlation analysis (sig. 1-tailed) - p≤0.05

**Table 3:** Associations between clinical (PI, GI and PD) and immunological results for children with gingival bleeding, considering NDM and DM groups

	Condition	PID	PIP	PIT	GID	GIP	GIT	PDD	PDP	PDT
1L-1β	NDM	r=-0.2368	r=-0.1314	r=-0.0873	r=0.3629	r=0.2810	r=0.3969	r=0.1170	r=0.5629	r=0.3910
		p=0.459	p=0.684	p=0.787	p=0.246	p=0.376	p=0.201	p=0.717	p=0.057	p=0.209
	DM	r=0.4102	r=0.5387	r=0.5664	r=0.2995	r=0.2363	r=0.2847	r=-0.1141	r=0.2819	r=0.0619
		p=0.361	p=0.212	p=0.185	p=0.514	p=0.610	p=0.536	p=0.807	p=0.540	p=0.895
TNF-α	NDM	r=0.3277	r=0.0482	r=0.3740	r=0.0010	r=-0.5255	r=-0.3758	r=-0.2531	**r=-0.7000**	r=-0.5589
		p=0.298	p=0.882	p=0.231	p=0.998	p=0.079	p=0.229	p=0.427	p=0.011*	p=0.059
	DM	**r=0.9346**	r=0.6450	**r=0.8011**	**r=0.7719**	r=0.3044	r=0.5176	r=0.0570	r=0.0382	r=0.0523
		p=0.002*	p=0.118	p=0.030*	p=0.042*	p=0.507	p=0.234	p=0.903	p=0.935	p=0.911
IL-6	NDM	r=-0.2206	r=-0.2787	r=-0.3017	r=-0.1404	r=-0.1070	r=-0.1654	r=-0.2270	r=-0.3676	r=-0.3570
		p=0.491	p=0.380	p=0.341	p=0.663	p=0.741	p=0.607	p=0.478	p=0.240	p=0.255
	DM	r=0.9227	r=0.6076	**r=0.7789**	r=0.5371	r=0.0501	r=0.2591	r=0.0313	r=-0.0642	r=-0.0109
		p=0.003*	p=0.148	p=0.039*	p=0.214	p=0.915	p=0.575	p=0.947	p=0.891	p=0.982

PID = Plaque index for deciduous teeth, PIP = Plaque index for permanent teeth, PIT = Plaque index for all teeth, GID = Gingival index for deciduous teeth, GIP = Gingival index for permanent teeth, GIT=Gingival index for all teeth. PDD = probing depth for deciduous teeth, PDP= probing depth for permanent teeth, PDT=probing depth for all teeth.

* Positive correlation obtained by the Pearson correlation analysis (sig. 1-tailed) - p≤0.05

### Microbiological profile

**Figure 1:**
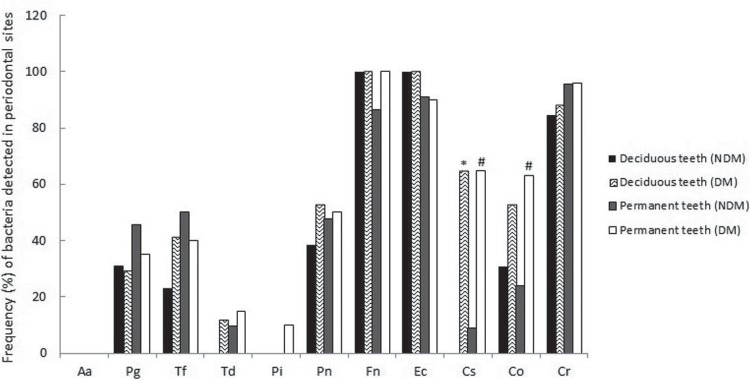
Frequency of bacteria detected on crevicular gingival fluid of children with gingival bleeding (GI ≥ 2), considering deciduous and permanent teeth. *Aggregatibacter actinomycetemcomitans* (Aa), *Porphyromonas gingivalis* (Pg), *Tannerella forsythia* (Tf), *Treponema denticola* (Td), *Prevotella intermedia* (Pi), *Prevotella nigrescens* (Pn), *Fusobacterium nucleatum* (Fn), *Eikenella corrodens* (Ec), *Capnocytophaga sputigena* (Cs), *Capnocytophaga ochracea* (Co), *Campylobacter rectus* (Cr).

Figure 1 and Table 4 show relative and absolute frequencies of periodontal bacteria, as well as each species combination, detected in the crevicular gingival fluid of DM and NDM children, considering deciduous and permanent teeth and GI ≥2. The DM group presented statistically higher levels of *Capnocytophaga sputigena* for both dentitions and *Capnocytophaga ochracea* for permanent dentition when compared to the NDM group. *Prevotella intermedia* was detected in only two DM patients and *Aggregatibacter actinomycetemcomitans* was not detected in any children in this study. *Fusobacterium nucleatum* and *Campylobacter rectus* were the most prevalent bacteria, followed by *Eikenella corrodens*, in both populations. Bacteria from the “red complex” were detected in few sites of both the DM and NDM groups. The best combination of “orange complex” pathogens was *Fusobacterium nucleatum* and *Campylobacter rectus.* However, both of them combined with *Prevotella nigrescens* harbored around 42% and 50% of NDM and DM groups sites, respectively. The “green complex” – represented by the combination of *Eikenella corrodens*, *Capnocytophaga sputigena* and *Capnocytophaga ochracea* in this study – was definitely more prevalent in the periodontal sites of DM children. Even with the inclusion of *Campylobacter rectus* in the “green complex”, this result did not change.

* Significant difference between DM and NDM (in deciduous teeth), according to the Chi square (χ2) test. (p≤0.05).

# Significant difference between DM and NDM (in permanent teeth), according to the Chi square (χ2) test. (p≤0.05).

**Table 4:** Absolute and relative frequency values - n (%) - of bacteria combinations detected on crevicular gingival fluid of children with gingival bleeding, considering deciduous and permanent teeth

		Deciduous teeth		Permanent teeth		Total	
		NDM (13 sites)	DM (17 sites)	NDM (22 sites)	DM (20 sites)	NDM (35 sites)	DM (37 sites)
Red complex	Pg+Tf	1(7.7)	2 (11.8)	5 (22.7)	5 (25)	6 (17.2)	7 (18.9)
	Pg+Td	0	0	0	1 (5)	0	1 (2.7)
	Tf+Td	0	2 (11.8)	1(4.5)	2 (10)	1 (2.8)	4 (10.8)
	Pg+Tf+Td	0	0	0	1 (5)	0	1 (2.7)
Orange complex	Pi+Fn	0	0	0	2 (10)	0	2 (5.8)
	Pi+Pn	0	0	0	0	0	0
	Pi+Cr	0	0	0	2	0	2 (5.8)
	Fn+Pn	5 (38.4)	9 (52.9)	10 (45.5)	10 (50)	15(42.8)	19 (51.4)
	Fn +Cr	11 (84.6)	15 (88.2)	21 (95.5)	19(95	32 (91.4)	34 (91.9)
	Cr+Pn	5 (38.4)	8 (47.1)	10(45.5)	10 (50)	15 (42.9)	18 (48.6)
	Pi+Fn+Pn	0	0	0	0	0	0
	Pi+Fn+Pn+Cr	0	0	0	0	0	0
Green complex	Ec+Cs	0*	11 (64.7)	2 (9)*	10 (50)	2 (5.7)*	21 (56.8)
	Ec+Co	4 (30.7)	9 (52.9)	4 (18.1)*	10 (50)	8 (22.9)*	19 (51.4)
	Cs+Co	0*	9 (52.9)	1 (4.5)*	9 (45)	1 (2.8)*	18 (48.6)
	Ec+Cs+Co	0*	9 (52.9)	1 (4.5)*	7 (35)	1 (2.8)*	16 (43.2)
Green complex (+Cr)	Ec+Cr	11 (84.6)	15 (88.2)	21 (95.5)	17 (85)	32 (91.4)	32 (86.5)
	Co+Cr	4 (30.7)	7 (41.1)	4 (18.1)*	12 (60)	8 (22.9)*	19 (51.4)
	Cs+Cr	0*	9 (52.9)	2 (9)*	13 (65)	2 (5.4)*	22 (59.5)
	Ec+Cs+Cr	0*	9 (52.9)	2 (9)*	11 (55)	2 (5.4)*	20 (54.0)
	Ec+Co+Cr	4 (30.7)	7 (41.1)	2 (9)*	10 (50)	6 (17.1)*	17 (45.9)
	Cs+Co+Cr	0*	7 (41.1)	1 (4.5)*	9 (45)	1 (2.8)*	16 (43.2)
	(Ec+Cs+Co)+ Cr	0*	7(41.1)	1 (4.5)*	7 (35)	1 (2.8)*	14 (37.8)

* Significant difference between DM and NDM, according to the Chi square (χ2) test. (p≤0.05).

## Discussion

Most studies on the topic have concluded that DM children have higher levels of gingival inflammation when compared to NDM patients, suggesting that diabetes is an aggravating factor for periodontal disease^1,15,19,31^. However, in this study, children showed good gingival health, which is demonstrated by the low percentage of scores above 2 in Plaque Index and Gingival Index and reduced probing depths (average of approximately 1.5 mm), in both dentitions, regardless of the presence of diabetes mellitus. These results are in agreement with the data obtained by Sbordone, et al.^25^ (1998) who observed that, even after 3 years of monitoring, no differences were detected in the periodontal evaluation of patients with type 1 diabetes mellitus when compared to patients without diabetes^25^. A limitation of this study is the sample size that could have reduced the probability to detect difference between groups. Moreover, when comparing periodontal studies, differences may be detected in the clinical examinations related to recording design, type/number of sites assessed and the periodontal probe used to measure periodontal disease^15^. The protocol used in this study (four periodontal sites) showed the smallest bias and highest sensitivity of prevalence estimates amongst other tested protocols, according to Susin, et al.^27^ (2005). Additionally, good oral hygiene habits, as presented in this study, helped to maintain the periodontal health of both groups. Other important factors that could also have influenced the clinical results of different study are the duration of diabetes and the glucose levels^1^. Al-Khabbaz, et al.^1^ (2013) observed that, in children with type 1 diabetes, periodontitis was significantly associated with longer duration of diabetes and older age at diagnosis of diabetes. Studies have demonstrated that poor metabolic control, including high levels of fasting glucose and glycosylated hemoglobin are important factors that could increase the susceptibility to periodontal disease, as well as other systemic complications of diabetes mellitus^3,22^.

Children and adolescents with uncontrolled glycemic control, represented by higher HbA1c concentrations or higher fasting glucose, had higher frequency of caries and gingivitis^22^. In this study, the levels of HbA1c found in DM children were statistically higher than those observed in the control children, but according to acceptable glycemic parameters, denoting low influence of glycemic control on periodontal parameters. Lim, et al.^9^ (2007) evaluated 181 adult patients with diabetes and studied the relationship between metabolic control markers and inflammation and the severity of periodontal disease in patients with diabetes mellitus. These authors found positive correlations between HbA1c and the percentage of sites with probing depths ≥5 mm, total cholesterol, LDL and triglycerides. Positive associations between periodontal status and lipid and immunological profiles were found in this study. Snell-Bergeon, et al.^24^ (2010) evaluated 553 patients with type 1 diabetes mellitus and 215 healthy patients, aged between 10 and 22 years, and observed that high levels of IL-6 and fibrinogen were associated with lipid profile and may collaborate with systemic complications in individuals with diabetes. The same was observed in our study. IL-6 was positively correlated with LDL, TC and TL for both groups. Although most of the lipid parameters were higher in DM children when compared to NDM children, statistical differences were observed only for HDL, TRG and TL. Larger samples could *increase* the chance of finding a *significant difference* for these parameters. Studies evaluating lipid parameters and periodontal disease in children or adolescents with diabetes were not found.

In this study, the prevalence of bacteria from the “red complex” (*Porphyromonas gingivalis, Tannerella forsythia* and *Treponema denticola*) was low and there was no difference between DM and NDM groups. These bacteria have been related to the pathogenesis of periodontal disease and are frequently found in patients with chronic periodontitis^7^. When considering the “orange complex”, *P. intermedia* was detected only in the permanent teeth of two patients*. Fusobacterium nucleatum* and *Prevotella nigrescens* were detected in about half of the evaluated sites on both dentitions for both DM and NDM subjects. One interesting result was the marked combination of *Fusobacterium nucleatum* and *Prevotella nigrescens* in the permanent dentition. Okuda, et al.^14^(2012) observed synergism between *Prevotella* species, including *Prevotella nigrescens*, and *Fusobacterium nucleatum*, in biofilm formation, suggesting that these Gram-negative bacteria in the subgingival crevice could play an important role in the development of chronic periodontitis.

*Campylobacter rectus* has been included in the “orange complex”, which is considered a secondary group of pathogens in periodontal infections^26^. However, one study has detected high levels of *Campylobacter rectus* associated with periodontal health^6^, as observed in this study. The “green complex” – represented by the combination of *Eikenella corrodens*, *Capnocytophaga sputigena* and *Capnocytophaga ochracea* – was definitely more prevalent in the periodontal sites of children with DM. Kimura, et al.^8^(2002) observed that some putative periodontal bacteria, such as *E. corrodens, A. actinomycetemcomitans, C. sputigena, C. ochracea* and *C. rectus,* colonize earlier in the oral cavity than other species also related to periodontal disease. Ciantar, et al.^4^ (2005) showed higher numbers of *Capnocytophaga* spp. in periodontitis sites of DM patients in comparison with healthy sites in DM and NDM adult patients. *C. ochracea* and *C. granulosa* were the most prevalent species. Sakalauskiene, et al.^20^ (2014) observed a strong relationship between the presence of *F. nucleatum* and *Capnocytophaga* spp. and poorer metabolic control in type 1 diabetes patients (HbA1c) and in all clinical parameters of periodontal pathology. Few studies have evaluated the microbiota from subgingival sites of children with type 1 diabetes mellitus, and no consistent data regarding the relationship between periodontal pathogens and diabetes were found^19,25^.

The inflammatory host response to an oral bacterial challenge is a critical determinant in the outcome of patient health or disease. Systemic inflammatory diseases, such as diabetes, alter the host environment and could increase the patient’s vulnerability to gingivitis due to changes in the inflammatory response to microbial challenges^12,19,31^. Gingival crevicular fluid is considered a good source of locally and systemically derived biomarkers of periodontal disease. However, the collection of gingival crevicular fluid in children is difficult because the gingival sulci in the primary teeth are shallower than in permanent teeth, and this biological fluid is produced at low rate. In this study, the immunological analyses did not show differences between IL-1β, TNF-α and IL-6 levels among DM and NDM children. However, a positive correlation between these serum biomarkers (IL-1b for NDM and TNF-α and IL-6 for DM) and gingival status was observed. Ulker, et al.^29^ (2008) observed higher levels of IL-1β and TNF-α in the gingival fluid of patients who had gingivitis when compared to those with a healthy periodontium. Those findings were positively correlated with probing depths and clinical attachment loss. Salvi, et al.^19^ (2010) found a high concentration of IL-1β and IL-8 in patients with type 1 diabetes when compared to healthy individuals in an experimental gingivitis method. Those authors also verified a correlation between this inflammatory biomarker and some bacterial species belonging to the “orange complex” in children with diabetes.

In conclusion, clinical and immunological profiles were similar between DM and NDM children. The presence of Capnocytophaga sputigena and Capnocytophaga ochracea was associated with gingivitis in DM children. Glycemic and lipid parameters were higher in patients with diabetes, but remained within normal values. Longitudinal clinical studies using larger patient groups are necessary to confirm the influence of diabetes on the microbiological and immunological parameters and their relationship with gingivitis in children.

## References

[1] Al-Khabbaz AK, Al-Shammari KF, Hasan A, Abdul-Rasoul M (2013). Periodontal health of children with type 1 diabetes mellitus in Kuwait: a case-control study. Med Princ Pract.

[2] Ashimoto A, Chen C, Bakker I, Slots J (1996). Polymerase chain reaction detection of 8 putative periodontal pathogens in subgingival plaque of gingivitis and advanced periodontitis lesions. Oral Microbiol Immunol.

[3] Carneiro VL, Fraiz FC, Ferreira FM, Pintarelli TP, Oliveira AC, Boguszewski MC (2015). The influence of glycemic control on the oral health of children and adolescents with diabetes mellitus type 1. Arch Endocrinol Metab.

[4] Ciantar M, Gilthorpe MS, Hurel SJ, Newman HN, Wilson M, Spratt DA (2005). Capnocytophaga spp. in periodontitis patients manifesting diabetes mellitus. J Periodontol.

[5] Conrads G, Mutters R, Fischer J, Brauner A, Lütticken R, Lampert F (1996). PCR reaction and dot-blot hybridization to monitor the distribution of oral pathogens within plaque samples of periodontally healthy individuals. J Periodontol.

[6] Cortelli SC, Cortelli JR, Aquino DR, Holzhausen M, Franco GC, Costa FO (2009). Clinical status and detection of periodontopathogens and Streptococcus mutans in children with high levels of supragingival biofilm. Braz Oral Res.

[7] Haffajee AD, Socransky SS (1994). Microbial etiological agents of destructive periodontal diseases. Periodontol 2000.

[8] Kimura S, Ooshima T, Takiguchi M, Sasaki Y, Amano A, Morisaki I (2002). Periodontopathic bacterial infection in childhood. J Periodontol.

[9] Lim LP, Tay FB, Sum CF, Thai AC (2007). Relationship between markers of metabolic control and inflammation on severity of periodontal disease in patients with diabetes mellitus. J Clin Periodontol.

[10] Loe H, Silness J (1963). Periodontal disease in pregnancy: prevalence and severity. Acta Odontol Scand.

[11] Matsson L, Goldberg P (1985). Gingival inflammatory reaction in children at different ages. J Clin Periodontol.

[12] Mealey BL, Rose LF (2008). Diabetes mellitus and inflammatory periodontal diseases. Compend Contin Educ Dent.

[13] Modéer T, Wondimu B (2000). Periodontal diseases in children and adolescents. Dent Clin North Am.

[14] Okuda T, Kokubu E, Kawana T, Saito A, Okuda K, Ishihara K (2012). Synergy in biofilm formation between Fusobacterium nucleatum and Prevotella species. Anaerobe.

[15] Orbak R, Simsek S, Orbak Z, Kavrut F, Colak M (2008). The influence of type-1 diabetes mellitus on dentition and oral health in children and adolescents. Yonsei Medical J.

[16] Page RC, Kornman KS (1997). The pathogenesis of human periodontitis: an introduction. Periodontol 2000.

[17] Preshaw PM (2008). Host response modulation in periodontics. Periodontol 2000.

[18] Rotimi VO, Salako NO, Divia M, Asfour L, Kononen E (2010). Prevalence of periodontal bacteria in saliva of Kuwaiti children at different age groups. J Infect Public Health.

[19] Salvi GE, Franco LM, Braun TM, Lee A, Rutger Persson G, Lang NP (2010). Pro-inflammatory biomarkers during experimental gingivitis in patients with type 1 diabetes mellitus: a proof-of-concept study. J Clin Periodontol.

[20] Sakalauskiene J, Kubilius R, Gleiznys A, Vitkauskiene A, Ivanauskiene E, Šaferis V (2014). Relationship of clinical and microbiological variables in patients with type 1 diabetes mellitus and periodontitis. Med Sci Monit.

[21] Sardi JC, Duque C, Camargo GA, Hofling JF, Gonçalves RB (2011). Periodontal conditions and prevalence of putative periodontopathogens and Candida spp. in insulin-dependent type 2 diabetic and non-diabetic patients with chronic periodontitis: a pilot study. Arch Oral Biol.

[22] Seckin D, Ilhan N, Ertugrul S (2006). Glycaemic control, markers of endothelial cell activation and oxidative stress in children with type 1 diabetes mellitus. Diabetes Res Clin Pract.

[23] Silness J, Loe H (1964). Periodontal disease in pregnancy. II. Correlation between oral hygiene and periodontal condition. Acta Odontol Scand.

[24] Snell-Bergeon JK, West NA, Mayer-Davis EJ, Liese AD, Marcovina SM, D'Agostino RB (2010). Inflammatory markers are increased in youth with type 1 diabetes: the SEARCH Case-Control study. J Clin Endocrinol Metab.

[25] Sbordone L, Ramaglia L, Barone A, Ciaglia RN, Iacono VJ (1998). Periodontal status and subgingival microbiota of insulin-dependent juvenile diabetics: a 3-year longitudinal study. J Periodontol.

[26] Socransky SS, Haffajee AD, Cugini MA, Smith C, Kent RL (1998). Microbial complexes in subgingival plaque. J Clin Periodontol.

[27] Susin C, Kingman A, Albandar JM (2005). Effect of partial recording protocols on estimates of prevalence of periodontal disease. J Periodontol.

[28] Suzuki N, Yoshida A, Saito T, Kawada M, Nakano Y (2004). Quantitative microbiological study of subgingival plaque by real-time PCR shows correlation between levels of Tannerella forsythensis and Fusobacterium spp. J Clin Microbiol.

[29] Ulker AE, Tulunoglu O, Ozmeric N, Can M, Demirtas S (2008). The evaluation of cystatin C, IL-1beta, and TNF-alpha levels in total saliva and gingival crevicular fluid from 11- to 16-year-old children. J Periodontol.

[30] Watanabe K, Frommel TO (1996). Porphyromonas gingivalis, Actinobacillus actinomycetemcomitans and Treponema denticola detection in oral plaque samples using the polymerase chain reaction. J Clin Periodontol.

[31] Xavier AC, Silva IN, Costa FO, Corrêa DS (2009). Periodontal status in children and adolescents with type 1 diabetes mellitus. Arq Bras Endocrinol Metabol.

